# Interobserver and intraobserver agreement for gastric mucosa atrophy

**DOI:** 10.1186/s12876-015-0327-x

**Published:** 2015-08-04

**Authors:** Tomohiro Miwata, Duc Trong Quach, Toru Hiyama, Rika Aoki, Huy Minh Le, Phuong Luu Ngoc Tran, Masanori Ito, Shinji Tanaka, Koji Arihiro, Naomi Uemura, Kazuaki Chayama

**Affiliations:** 1 Department of Gastroenterology and Metabolism, Graduate School of Biomedical Sciences, Hiroshima University, Hiroshima, Japan; 2 Department of Endoscopy, University Medical Center, Ho Chi Minh, Vietnam; 3 Health Service Center, Hiroshima University, Higashihiroshima, Japan; 4 Department of Internal Medicine, Tokushima Health Screening Center, Tokushima, Japan; 5 Department of Endoscopy, Hiroshima University Hospital, Hiroshima, Japan; 6 Department of Pathology, Hiroshima University Hospital, Hiroshima, Japan; 7 Department of Gastroenterology and Hepatology, National Center for Global Health and Medicine, Ichikawa, Japan

## Abstract

**Background:**

The grade of gastric mucosa atrophy caused by *Helicobacter pylori* (*H. pylori*) infection is closely associated with the risk of gastric cancer, especially of the intestinal type. Interobserver and intraobserver agreement for endoscopic gastric mucosa atrophy in subjects with *H. pylori*-uninfected, currently infected and past infected was investigated.

**Methods:**

Endoscopic images of 91 patients, 34 images per patient, were assessed. The assessors were 4 endoscopist groups: Japanese and Vietnamese experienced (≥7, ≤ 15 year experience with endoscopy) and Japanese and Vietnamese beginner (≤ 3 year experience) groups. Each group comprised 3 endoscopists. The grades of atrophy were classified as 3: none to mild (C-0 and C-1), moderate (C-2 and C-3), and severe (O-1, O-2, and O-3) using the Kimura-Takemoto Classification. After a period of 2 weeks, images of all patients were reevaluated by the investigators. Interobserver and intraobserver agreement was calculated by kappa statistics.

**Results:**

The kappa values for the interobserver agreement in the groups of Japanese and Vietnamese experienced, and Japanese and Vietnamese beginner were 0.474, 0.408, 0.291, and 0.373, respectively. The kappa value of intraobsever agreement in the Japanese and Vietnamese experienced endoscoists ranged from 0.585 to 0.871. On the other hand, the value in the beginner endoscopists ranged wider than that in experienced endoscopists, from 0.264 to 0.866.

**Conclusions:**

Our results indicated that, although intraobserver agreement for gastric mucosa atrophy was good to excellent, interobserver agreement was moderate in experienced endoscopists. This suggests that better guidelines and firm criteria may be needed to properly diagnose and grade gastric atrophy.

## Background

Chronic gastritis is a condition mostly caused by *Helicobacter pylori* (*H. pylori*) infection [1]. An important stage in the multistep process leading to this condition is the development of atrophy of the gastric mucosa. Subjects infected with *H. pylori* may first develop non-atrophic, antral-predominant gastritis, a condition associated with duodenal ulcer, and then atrophic gastritis, a condition associated with gastric ulcer and cancer. The grade of gastric mucosa atrophy is closely associated with the risk of gastric cancer, especially of the intestinal type. The relative risk of gastric cancer is 1.7 in subjects with moderate gastric mucosa atrophy and 4.9 in those with severe gastric mucosa atrophy compared with those with no or mild gastric mucosa atrophy [2].

To assess the gastric mucosa atrophy histopathologically, however, the Operative Link for Gastritis Assessment (OLGA) Staging System was published in 2007 [3]. We previously examined the correlation between endoscopic gastric mucosa atrophy assessed by the Kimura-Takemoto Classification system [4] and histopathologic gastric mucosa atrophy assessed by the OLGA Staging System [5]. The result was that high-stage OLGA gastritis and extensive intestinal metaplasia with incomplete subtype were clustered in patients with endoscopic gastric mucosa atrophy rated as moderate-to-severe.

Several researchers have reported interobserver and intraobserver agreement in the histopathologic assessment of *H. pylori*-associated chronic gastritis [6–8]. However, there have been no studies on interobserver and intraobserver agreement in the endoscopic assessment of gastric mucosa atrophy. This agreement may be crucial to estimate the risk of gastric cancer. We therefore examined interobserver and intraobserver agreement for endoscopic gastric mucosa atrophy in subjects with *H. pylori*-uninfected, currently infected and past infected, *i.e.*, eradicated, in 12 assessors from 2 Asian countries, Japan, where endoscopy has already been widely applied, and Vietnam, where endoscopy is now spreading.

## Methods

### Patients

A total of 91 patients who underwent esophagogastroduodenoscopy (EGD) in Hiroshima University Hospital (Hiroshima, Japan) in August 2013 were prospectively enrolled in this study. Patients with gastric localized lesions such as gastric ulcer and cancer, those with a previous history of gastric surgery, and those who received proton pump inhibitors within 1 month prior to the study were excluded. Patients were divided into 3 groups: the *H. pylori*-uninfected group, *i.e.*, those who had never been infected with *H. pylori*, those with current infection, and the *H. pylori*-past infected group, *i.e.*, those with previously treated infection. *H. pylori* status was examined with at least 2 of the following 4 examinations: histologic examination of 2 biopsies, one from the antrum and the other from the corpus; serum and urine *H. pylori* antibody; and ^13^C-urea breath test. Patients with at least one positive test were regarded as having current *H. pylori* infection. Patients with all tests negative and no history of *H. pylori* eradication treatment were regarded as *H. pylori*-uninfected. Patients with all tests negative and history of *H. pylori* eradication treatment were regarded as *H. pylori*-past infected.

### Endoscopic images

Stored images were retrieved from the computerized database of the Department of Endoscopy, Hiroshima University Hospital by use of a digital image filing system, Nexus (Fujifilm Co., Ltd., Tokyo, Japan). The size per endoscopic image was approximately 500 kb. These images were obtained during the EGD procedure with a GIF-H260 or GIF-H260Z endoscope (Olympus Optical Co., Ltd., Tokyo, Japan), after obtaining the written informed consent of the patient. The standard image series consisted of 34 images: 5 of the prepylorus and antrum, 4 of the angulus, 21 of the corpus (9 look-ups and 12 look-downs), and 4 of the cardia and fornix. The data were presented to the investigators as a PowerPoint presentation (Microsoft, Redmond, WA, USA) and were assessed on a 17-inch standard-definition monitor.

### Assessors

The assessors were divided into 4 endoscopist groups: Japanese and Vietnamese experienced (≥ 7, ≤ 15 year experience with endoscopy) and Japanese and Vietnamese beginner (≤ 3 year experience) groups. Each group comprised 3 endoscopists.

### Assessment of images

**Fig. 1:**
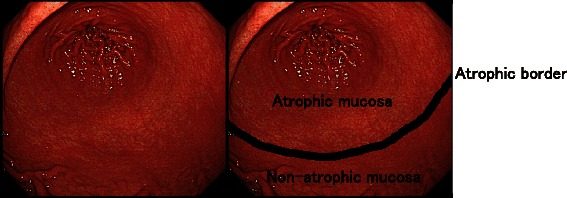
Representative example of a gastric atrophic border. The atrophic border is the boundary between the pyloric and fundic gland territories, which is endoscopically recognized by discriminating differences in the color and height of the gastric mucosa

**Fig. 2:**
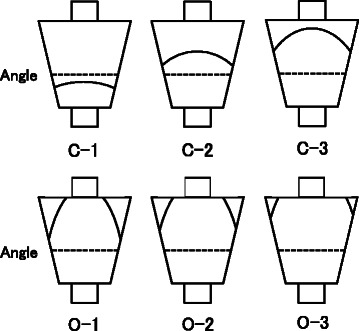
Classification of gastric mucosa atrophy (Kimura-Takemoto Classification). Cases of closed-type gastric mucosa atrophy have an atrophic boundary between the fundic mucosa and the pyloric mucosa in the antrum or lesser curvature of the gastric body. Cases of open-type gastric mucosa atrophy have an atrophic boundary in the lateral wall or greater curvature of the gastric body. C, closed; O, open

All images of the 91 patients were evaluated by the 4 endoscopist groups. All endoscopists were blinded to the patients’ clinical information. Each investigator assessed endoscopic gastric mucosa atrophy using the Kimura-Takemoto Classification [4]. The atrophic border is defined as the boundary between the pyloric and fundic gland regions, which is endoscopically recognized by the difference in color and height of the gastric mucosa between the two sides of the border (Figs. 1 and 2). The grades of atrophy were reclassified into 3: none to mild (C-0 and C-1), moderate (C-2 and C-3), and severe (O-1, O-2, and O-3).

After a period of 2 weeks had passed, the images of all 91 patients were reevaluated by the assessors, and the grades of gastric mucosa atrophy were recorded in a similar fashion. These results were used for the calculation of intraobserver agreement (kappa value).

### Statistical analysis

The interobserver and intraobserver agreements were calculated by kappa statistics [9]. The agreement results were defined as follows: poor, ≤ 0.2; mild, 0.2 to 0.4; moderate, 0.4 to 0.6; good, 0.6 to 0.8, and excellent, 0.8 to 1.

### Ethical approval

This study was approved by the Ethical Committee of Hiroshima University (Eki-846).

## Results

### Patients

All 91 patients were Japanese with a mean age of 62.5 (standard deviation, 13.4) years, and the male:female ratio was 34:57 (Table 1). Of the patients, 72 were those with untreated *H. pylori* (24 had never been infected and 48 were currently infected at the time of examination), and 19 were those with *H. pylori*-past infected.

**Table 1:** Patients’ characteristics

Characteristics	All (*n* = 91)	*H. pylori*-uninfected (*n* = 24)	*H. pylori*-currently infected (*n* = 48)	*H. pylori*-past infected (*n* = 19)
Age (y) (mean, SD^*^)	62.5, 13.8	53.2, 12.9	63.3, 11.1	73.3, 11.8
Male/female	49/42	13/11	25/23	11/8

*SD, standard deviation

### Assessors

The mean periods of endoscopic experience of the Japanese experienced (Y.U., Y. S., and T. M.) and the Vietnamese experienced (Q.D.T., L.H.M., and T.P.L.N.) and Japanese beginner (H.K., E.S., and M.H.) and Vietnamese beginner (L.Q.D., V.N.H.T., and N.K.T.N) groups were 9.7, 10.7, 1.7, and 1.7 years, respectively.

### Concordance rate for diagnosis of gastric mucosa atrophy by EGD and *H. pylori* state

Concordance rate for diagnosis of gastric mucosa atrophy by EGD and *H. pylori* state, *i.e.*, none to mild gastric atrophy and *H. pylori*-uninfected, and moderate to severe gastric atrophy and *H. pylori*-currently or past infected, was assessed in each investigator. The rate in experienced endoscopists ranged from 81.3 % to 92.3 % (Table 2). On the other hand, the rate in beginner endoscopists ranged from 70.3 % to 90.1 %, wider than that in experienced endoscopists.

**Table 2:** Concordance rate for diagnosis of gastric atrophy by EGD and *H. pylori* state in each assessor

Assessor	Experience (y)	Concordance rate (%)
Japanese experienced No. 1	10	92.3
Japanese experienced No. 2	10	88.0
Japanese experienced No. 3	9	86.8
Vietnamese experienced No. 1	10	90.1
Vietnamese experienced No. 2	7	81.3
Vietnamese experienced No. 3	15	86.8
Japanese beginner No. 1	3	90.1
Japanese beginner No. 2	1	73.6
Japanese beginner No. 3	1	84.6
Vietnamese beginner No. 1	2	87.9
Vietnamese beginner No. 2	2	70.3
Vietnamese beginner No. 3	1	83.5

### Grade of gastric mucosa atrophy in patients assessed

According to the first evaluation by the Japanese experienced assessor No. 1, who had the highest concordance rate for diagnosis of gastric mucosa atrophy by EGD and *H. pylori* state in the present study, the number of subjects with none to mild (C-0 and C-1) atrophy was 29, that with moderate (C-2 and C-3) was 33, and that with severe (O-1, O-2, and O-3) atrophy was 29.

### Interobserver agreement

The Fleiss kappa values for the interobserver agreement for endoscopic gastric mucosa atrophy in the 4 groups are listed in Table 3. In all 91 patients, the kappa values in the Japanese and Vietnamese experienced, and Japanese and Vietnamese beginner groups were 0.4738, 0.408, 0.2914, and 0.3730, respectively. The agreement in the experienced groups was moderate, whereas that in the beginner groups was mild.

**Table 3:** Kappa value for the interobserver agreement for gastric mucosa atrophy in each group

Assessor group^a^		All (*n* = 91)	*H. pylori*-uninfected (*n* = 24)	*H. pylori*-currently infected (*n* = 48)	*H. pylori*-past infected (*n* = 19)
Japanese experienced	Kappa value	0.474	0.583	0.295	0.401
	(95 % CI)	(0.390-0.558)	(0.352-0.814)	(0.151-0.438)	(0.212-0.589)
Vietnamese experienced	Kappa value	0.408	0.458	0.344	0.141
	(95 % CI)	(0.323-0.493)	(0.232-0.684)	(0.208-0.479)	(-0.044-0.326)
Japanese beginner	Kappa value	0.291	0.375	0.275	0.136
	(95 % CI)	(0.207-0.376)	(0.201-0.549)	(0.146-0.403)	(-0.051-0.322)
Vietnamese beginner	Kappa value	0.373	0.416	0.255	0.205
	(95 % CI)	(0.289-0.457)	(0.190-0.642)	(0.133-0.376)	(0.021-0.390)

^a^Each assessor group consists of 3 assessors

Among the 3 *H. pylori* states, uninfected, currently and past infected, the interobserver agreement in *H. pylori*-uninfected, was the highest in each group.

### Intraobserver agreement

The kappa values for the intraobserver agreement for gastric mucosa atrophy in the 4 groups are shown in Table 4. Two of the 3 Japanese experienced endoscopists showed excellent agreement, and all 3 of the Vietnamese experienced endoscopists showed good agreement. However, the agreement in Japanese and Vietnamese beginner endoscopists varied from fair to excellent.

**Table 4:** Kappa value for the intraobserver agreement for gastric mucosa atrophy in each assessor

Assessor		All (*n* = 91)	*H. pylori*-uninfected (*n* = 24)	*H. pylori*-currently infected (*n* = 48)	*H. pylori*-past infected (*n* = 19)
Japanese experienced No. 1	Kappa value	0.801	0.791	0.658	0.826
	(95 % CI)	(0.656-0.947)	(0.6277-0.9547)	(0.410-0.905)	(0.496-1.157)
Japanese experienced No. 2	Kappa value	0.585	0.601	0.351	0.521
	(95 % CI)	(0.439-0.730)	(0.4369-0.7649)	(0.122-0.580)	(0.201-0.841)
Japanese experienced No. 3	Kappa value	0.871	0.862	0.874	0.906
	(95 % CI)	(0.718-1.025)	(0.6918-1.0321)	(0.591-1.157)	(0.533-1.279)
Vietnamese experienced No. 1	Kappa value	0.680	0.625	0.452	0.613
	(95 % CI)	(0.530-0.831)	(0.225-1.025)	(0.180-0.723)	(0.222-1.008)
Vietnamese experienced No. 2	Kappa value	0.740	0.630	0.677	0.820
	(95 % CI)	(0.589-0.891)	(0.230-0.130)	(0.465-0.888)	(0.484-1.156)
Vietnamese experienced No. 3	Kappa value	0.728	0.495	0.716	0.666
	(95 % CI)	(0.581-0.875)	(0.095-0.895)	(0.481-0.950)	(0.353-0.997)
Japanese beginner No. 1	Kappa value	0.664	0.703	0.514	0.501
	(95 % CI)	(0.517-0.810)	(0.5388-0.8678)	(0.294-0.733)	(0.170-0.832)
Japanese beginner No. 2	Kappa value	0.429	0.485	0.324	0.195
	(95 % CI)	(0.273-0.585)	(0.3112-0.6583)	(0.110-0.538)	(-0.163-0.554)
Japanese beginner No. 3	Kappa value	0.264	0.261	0.261	0.275
	(95 % CI)	(0.118-0.410)	(0.0961-0.4248)	(0.039-0.484)	(-0.045-0.596)
Vietnamese beginner No. 1	Kappa value	0.714	0.630	0.753	0.346
	(95 % CI)	(0.563-0.866)	(0.230-0.130)	(0.520-0.986)	(0.023-0.669)
Vietnamese beginner No. 2	Kappa value	0.531	0.792	0.575	0.188
	(95 % CI)	(0.373-0.688)	(0.392-1.192)	(0.368-0.781)	(-0.170-0.546)
Vietnamese beginner No. 3	Kappa value	0.866	1.000	0.725	1.000
	(95 % CI)	(0.720-1.012)	(1.000-1.000)	(0.508-0.942)	(0.674-1.326)

Among the 3 *H. pylori* states, uninfected, currently and past infected, the highest intraobserver agreement differed assessor by assessor.

## Discussion

In the present study, we demonstrated that, although intraobserver agreement for gastric mucosa atrophy was good to excellent, interobserver agreement was moderate in experienced endoscopists. This suggests that better guidelines and firm criteria may be needed to properly diagnose and grade gastric atrophy. Truly, this is the first study on interobserver and intraobserver agreement for gastric mucosa atrophy by endoscopy.

To assess gastric mucosa atrophy endoscopically, the Kimura-Takemoto Classification was published in 1969 [4]. Since then, no other classification systems have been published, and the Kimura-Takemoto Classification is commonly used not only in research field^1,2^ but also in daily practice, especially in East and Southeast Asian countries, including Japan and Vietnam. The classification focuses on the identification of the endoscopic atrophic border and its location in the stomach. The original classification consists of 7 grades, and the complication has been pointed out. Therefore, in the present study, 3 grades, none to mild, moderate, and severe, was used.

Several researchers have reported interobserver and intraobserver agreement in the histopathologic assessment of *H. pylori*-associated chronic gastritis [6–8]. Although interobserver agreement among pathologists for intestinal metaplasia is good to excellent, that for glandular atrophy is mild to moderate according to these studies. On the other hand, the interobserver agreement among endoscopists for gastric mucosa atrophy was moderate in experienced endoscopists in the present study. The agreement was a quite similar result to that for glandular atrophy among pathologists. In addition, it has been reported that interobserver agreement for glandular atrophy among experienced gastrointestinal pathologists (moderate) was better than that among less-experienced pathologists (mild) [6]. In the present study, a similar result was obtained: interobserver agreement for gastric atrophy was higher in the experienced group than in the beginner group, in both the Japanese and Vietnamese endoscopists. These results suggest that this classification requires years of training to master well.

There has been one report that examined the relationship between OLGA and the Operative Link for Gastric Intestinal Metaplasia (OLGIM) Staging and endoscopic findings [10]. In the study, narrow-band imaging magnifying endoscopy was used. Degree of correspondence between endoscopic scores and histopathologic scores was 69.1 % for the antrum and 72.7 % for the corpus, *i.e.*, good correspondence. To grade gastric mucosa atrophy histopathologically, gastric biopsy samples are needed. Biopsy could cause massive bleeding. In addition to the bleeding risk, the histopathologic grading costs a lot compared with endoscopic grading does. Thus, endoscopic grading of gastric mucosa atrophy may be more beneficial than histopathologic grading. Examining the interobserver and intraobserver agreement for gastric mucosa atrophy by endoscopy is of great interest.

A good interobserver agreement could not be reached both in Japan and Vietnam, although good to excellent intraobserver agreement could be, in the present study. Of course, a perfect agreement by endoscopists was impossible because assessments of gastric mucosa atrophy were based on their subjects. However, better agreement may be needed to increase the reproducibility of the endoscopic findings. The criteria for grading endoscopic gastric atrophy should be improved. Simpler, well-defined criteria will improve the agreement. The OLGA Staging System is the system to assess the gastric mucosa atrophy histopathologically. In the system, glandular atrophy of the antrum and the corpus is evaluated, then, the stage is settled. Similar endoscopic system may work well.

Recently, *H. pylori* eradication treatment has been widely applied. Edema, rugal hyperplasia, and diffuse spotty erythema are characteristic endoscopic findings of *H. pylori*-associated chronic gastritis [11]. Significant changes in endoscopic findings of gastric mucosa after successful eradication appear as mottled patchy erythema [11]. Disappearance of spotty redness and the appearance of gastric flat erosion are also associated with successful eradication [12]. These changes may be caused by vanishing of the inflammation of the gastric mucosa. We hypothesized that the assessment of gastric atrophy would be easier after eradication because of the vanishing of the inflammation. However, the change of intraobserver agreement for atrophy before and after eradication differed by endoscopist and endoscopist: 7 increased and 5 decreased after eradication. Our hypothesis may be denied.

There are several limitations in the present study. First, the number of assessors is limited. These assessors may not be representative of experienced and beginner endoscopists. However, each group consisted of 3 assessors, and not 2 assessors. Our results may be more accurate compared with a group comprising only 2 assessors. Similar studies should be done to verify our research results. Second, the number of images assessed is relatively limited. If more images were assessed, more detailed findings could be obtained. Third, there could be false-positives or false-negatives in the diagnosis of *H. pylori* status. We adopted at least 2 methods for diagnosis; however, the possibilities for misdiagnosis cannot be denied. Finally, information on the period after *H. pylori* eradication was not available in all subjects because in some, *H. pylori* had been eradicated unintentionally, and some had forgotten when the eradication treatment was performed. As a next investigation, we are planning to assess endoscopic gastric mucosa atrophy using 3 image sets from identical subjects, for example, before, 1, and 3 years after eradication. With the investigation, we may be able to clarify in detail the changes of interobserver and intraobserver agreement after eradication.

## Conclusions

We demonstrated that interobserver agreement for gastric mucosa atrophy was moderate in experienced endoscopists, suggesting that better guidelines and firm criteria may be needed to properly diagnose and grade gastric atrophy.

## Abbreviations

*H. pylori*: *Helicobacter pylori*
OLGA: Operative Link for Gastritis Assessment
EGD: Esophagogastroduodenoscopy
OLGIM: Operative Link for Gastric Intestinal Metaplasia
